# IPS-1 Is Essential for the Control of West Nile Virus Infection and Immunity

**DOI:** 10.1371/journal.ppat.1000757

**Published:** 2010-02-05

**Authors:** Mehul S. Suthar, Daphne Y. Ma, Sunil Thomas, Jennifer M. Lund, Nu Zhang, Stephane Daffis, Alexander Y. Rudensky, Michael J. Bevan, Edward A. Clark, Murali-Krishna Kaja, Michael S. Diamond, Michael Gale

**Affiliations:** 1 Department of Immunology, University of Washington School of Medicine, Seattle, Washington, United States of America; 2 Departments of Medicine, Molecular Microbiology, and Pathology and Immunology, Washington University School of Medicine, St. Louis, Missouri, United States of America; 3 Department of Microbiology, University of Washington School of Medicine, Seattle, Washington, United States of America; University of North Carolina, United States of America

## Abstract

The innate immune response is essential for controlling West Nile virus (WNV) infection but how this response is propagated and regulates adaptive immunity *in vivo* are not defined. Herein, we show that IPS-1, the central adaptor protein to RIG-I-like receptor (RLR) signaling, is essential for triggering of innate immunity and for effective development and regulation of adaptive immunity against pathogenic WNV. IPS-1^−/−^ mice exhibited increased susceptibility to WNV infection marked by enhanced viral replication and dissemination with early viral entry into the CNS. Infection of cultured bone-marrow (BM) derived dendritic cells (DCs), macrophages (Macs), and primary cortical neurons showed that the IPS-1-dependent RLR signaling was essential for triggering IFN defenses and controlling virus replication in these key target cells of infection. Intriguingly, infected IPS-1^−/−^ mice displayed uncontrolled inflammation that included elevated systemic type I IFN, proinflammatory cytokine and chemokine responses, increased numbers of inflammatory DCs, enhanced humoral responses marked by complete loss of virus neutralization activity, and increased numbers of virus-specific CD8+ T cells and non-specific immune cell proliferation in the periphery and in the CNS. This uncontrolled inflammatory response was associated with a lack of regulatory T cell expansion that normally occurs during acute WNV infection. Thus, the enhanced inflammatory response in the absence of IPS-1 was coupled with a failure to protect against WNV infection. Our data define an innate/adaptive immune interface mediated through IPS-1-dependent RLR signaling that regulates the quantity, quality, and balance of the immune response to WNV infection.

## Author Summary

West Nile virus (WNV) is a mosquito-transmitted RNA virus that has emerged in the Western hemisphere and is now the leading cause of arboviral encephalitis in the United States. However, the virus/host interface that controls WNV pathogenesis is not well understood. Previous studies have established that the innate immune response and interferon (IFN) defenses are essential for controlling virus replication and dissemination. In this study, we assessed the importance of the RIG-I like receptor (RLR) signaling pathway in WNV pathogenesis through analysis of mice lacking IPS-1, the central adaptor molecule of RLR signaling. Our studies revealed that IPS-1 is essential for protection against WNV infection and that it regulates processes that control virus replication and triggering of innate immune defenses. We found that IPS-1 plays an important role in establishing adaptive immunity through an innate/adaptive interface that elicits effective antibody responses and controls the expansion of regulatory T cells. Thus, RLRs are essential for pathogen recognition of WNV infection and their signaling programs help orchestrate immune response maturation, regulation of inflammation, and immune homeostasis that define the outcome of WNV infection.

## Introduction

West Nile virus (WNV) is a neurotropic flavivirus and is an emerging public health threat. Infection with WNV now constitutes the leading cause of mosquito-borne and epidemic encephalitis in humans in the United States [1]. WNV is enveloped and contains a single strand positive sense RNA genome of approximately 11 kb in length that encodes three structural (C, prM/M, and E) and seven non-structural proteins (NS1, NS2A, NS2B, NS3, NS4A, NS4B, and NS5). It cycles enzootically between birds and *Culex* mosquitoes, with humans infected as dead-end hosts. WNV infection has been modeled in inbred mice wherein infection and pathogenesis recapitulate many of the features of human infection (reviewed in [2]). Following subcutaneous inoculation, WNV replicates in dendritic cells (DCs) at the portal of entry and in the draining lymph node. A primary viremia develops and virus spreads to visceral organs including the spleen, where further amplification occurs, leading to central nervous system (CNS) dissemination and encephalitis. In humans, WNV causes an acute febrile illness that can progress to severe and sometimes lethal neuroinvasive disease, especially in the elderly and immunocompromised [3]. However, healthy young adults are also afflicted with severe neurological disease [4],[5],[6], indicating that virulence can occur independently of immune deficiencies or aging.

Intracellular innate immune defenses and the actions of type I interferon (IFN) provide a first-line of defense against virus infection and are essential for the control of WNV replication, dissemination, and neurovirulence [7]. Innate antiviral immune defenses are triggered through the recognition of conserved pathogen associated molecular pattern (PAMP) motifs within viral products by intracellular pathogen recognition receptor (PRR) proteins in infected cells. PRR signaling directs downstream activation of latent transcription factors, including NF-κB, interferon regulatory factor (IRF)-3 and IRF-7, in a cell type-specific manner to induce antiviral response programs that include expression of proinflammatory cytokines, chemokines, type I IFN, and interferon stimulated genes (ISGs) [7],[8],[9],[10]. The ISG products induced through autocrine and paracrine actions of IFN confer antiviral activity by limiting virus replication and cell-to-cell virus spread. Modulation of IFN signaling has been identified as a virulence feature of pathogenic strains of WNV [11],[12].

The RLRs, retinoic acid inducible gene-I (RIG-I) and melanoma differentiation antigen 5 (MDA5) [13],[14],[15],[16], are PRRs that play critical roles in triggering immune defenses against RNA virus infection, including WNV. RIG-I and MDA5 are cytosolic RNA helicases that contain an amino terminal tandem caspase activation and recruitment domain (CARD). Upon engaging RNA substrates, the RLRs undergo a conformational change and bind to the mitochondrial associated protein, interferon promoter stimulator-1 (IPS-1) through a CARD-CARD interaction, leading to IPS-1-dependent signaling of IFN production and expression of immune response genes [17],[18]. RLR signaling and IPS-1 function have an essential role in triggering IFN defenses during WNV infection of mouse embryo fibroblasts (MEFs) and human cell lines *in vitro*. Cells lacking either RIG-I or MDA5 were attenuated in their ability to generate an effective innate immune response to infection, whereas cells lacking both RIG-I and MDA5 or those deficient in IPS-1 alone were unable to respond to infection with WNV and related flaviviruses [19],[20],[21],[22]. Recent studies examined the role of another class of pattern recognition receptors, Toll like receptor (TLR)3 and TLR7, and show that these receptors are also important PRRs of WNV infection, as they play a role in signaling IFN production and an inflammatory response upon viral ligand recognition [23],[24],[25]. TLR3 has been shown to contribute to both enhancement and protection of CNS inflammation and neurovirulence of WNV *in vivo* [23],[24], while TLR7-dependent signaling was shown to be essential for directing proper immune cell homing to sites of WNV infection during the adaptive immune response *in vivo* [25].

Type I IFN, a major product of PRR signaling, has been shown to link innate and adaptive immune responses. However, the specific PRR pathways that mediate this during acute WNV infection have not been delineated nor has the RLR pathway been evaluated in this context. The quantity and quality of the innate and adaptive immune responses after infection must be carefully regulated to avoid aberrant inflammation and immunopathogenesis. Regulatory T (T_reg_) cells and inflammatory dendritic cell (DC) subsets regulate inflammation during acute virus infection through T cell suppression and by modulating the trafficking and inflammatory cytokine production of immune cells into infected tissues [26],[27],[28]. Thus, the level of local and peripheral T_reg_ cells, and the composition of local DC subsets that develop during WNV infection may determine immune control and WNV disease.

Here, we assessed the role of RLR signaling and IPS-1 in WNV infection and immunity. Our studies define IPS-1 as an essential modulator of immunity *in vivo* and demonstrate that IPS-1-dependent signaling orchestrates an innate/adaptive immune interface that regulates immune responses to effectively control WNV infection.

## Results

### RIG-I and IPS-1 are essential for protection against WNV infection

**Figure 1:**
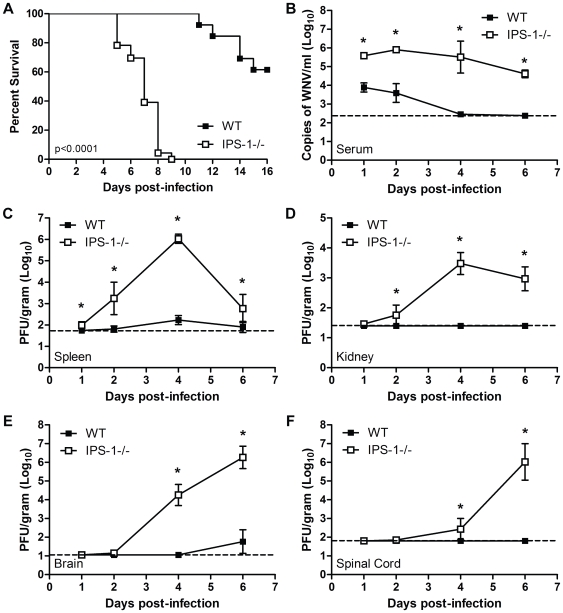
Virologic analysis in wild type and IPS-1^−/−^ mice. Adult wild type and IPS-1^−/−^ mice were infected s.c. with 100 PFU of WN-TX. (A) Differential lethality from WNV infection (WT n = 13; IPS-1^−/−^ n = 23; p<0.0001). B–F. Viral burden analysis of peripheral and CNS tissues from wild type and IPS-1^−/−^ mice infected s.c. with 100 PFU of WN-TX. (B) WNV RNA in serum and infectious virus in the (C) spleen, (D) kidney, (E) brain, and (F) spinal cord were determined by RT-qPCR assay (B) or viral plaque assay (C–F) of samples harvested on day 1, 2, 4, and 6 pi. Data are shown as copies of WNV genome RNA per ml of serum or PFU per gram of tissue for 3 to 6 mice per timepoint. Graphs show the mean +/− standard deviation for each measurement. Asterisk denotes p<0.05. The horizontal line indicates the lower limit of assay sensitivity.

WNV infection of primary embryonic fibroblasts recovered from RIG-I^−/−^ mice revealed that RIG-I was important in eliciting innate antiviral immune defenses early during infection, whereas MDA5 was important for enhancing and sustaining this response [21]. We further evaluated WNV infection of RIG-I^−/−^ or MDA5^−/−^ mice and confirmed that RIG-I serves a dominant role among the RLRs for the acute induction of innate immune defenses and protection against WNV infection *in vivo* (data not shown). Since the RLRs signal innate defenses through the IPS-1 adaptor protein [29], we also examined the role of IPS-1 in protection against WNV infection upon a sub-lethal virus challenge of wild type and IPS-1^−/−^ mice. IPS-1^−/−^ mice were highly susceptible to WNV infection and exhibited 100% mortality with an average survival time (AST) of 7.3 days as compared to wild type mice (38.5% mortality with an AST of 13.2 days; p<0.0001; **Fig 1A**). Thus, RIG-I and IPS-1-dependent signaling are essential for protection against WNV infection.

### IPS-1-dependent signaling controls WNV replication, tissue tropism, and CNS invasion

To define the role of IPS-1 in controlling WNV *in vivo*, wild type and IPS-1^−/−^ mice were infected subcutaneously (s.c.) with 100 PFU of WN-TX and viral burden within peripheral tissues and the CNS was measured over time post-infection (pi). IPS-1^−/−^ mice exhibited increased viremia compared to wild type mice (45.7 fold enhancement at day 1 pi, P<0.05) throughout the course of infection (**Fig 1B**). Similarly, viral loads in the spleen were elevated in the infected IPS-1^−/−^ mice (**Fig 1C**). WNV infection of IPS-1^−/−^ mice displayed an expanded tissue tropism as infectious virus was found in the kidneys, a tissue that is not normally permissive to infection in wild type mice (**Fig 1D**). WNV is typically detected in the CNS of wild type mice after s.c. challenge between 4 and 8 days pi [2]. Consistent with this time course, infected wild type mice exhibited detectable viral loads (average viral titer of 10^1.8^ pfu/gram of tissue) in the brain by day 6 p.i., although virus was not detected in the spinal cord (**Fig 1E and F**). In contrast, WNV spread to the brain (**Fig 1E**) and spinal cord of IPS-1^−/−^ mice (**Fig 1F**) by day 2 pi, with viral loads rising through day 6 pi. Together these results indicate that IPS-1, likely through RLR signaling of innate immune defenses, limits WNV replication, viremia, and peripheral spread, and is essential for the control of viral invasion of the CNS.

### IPS-1 regulates the innate immune response to WNV infection in myeloid cells

**Figure 2:**
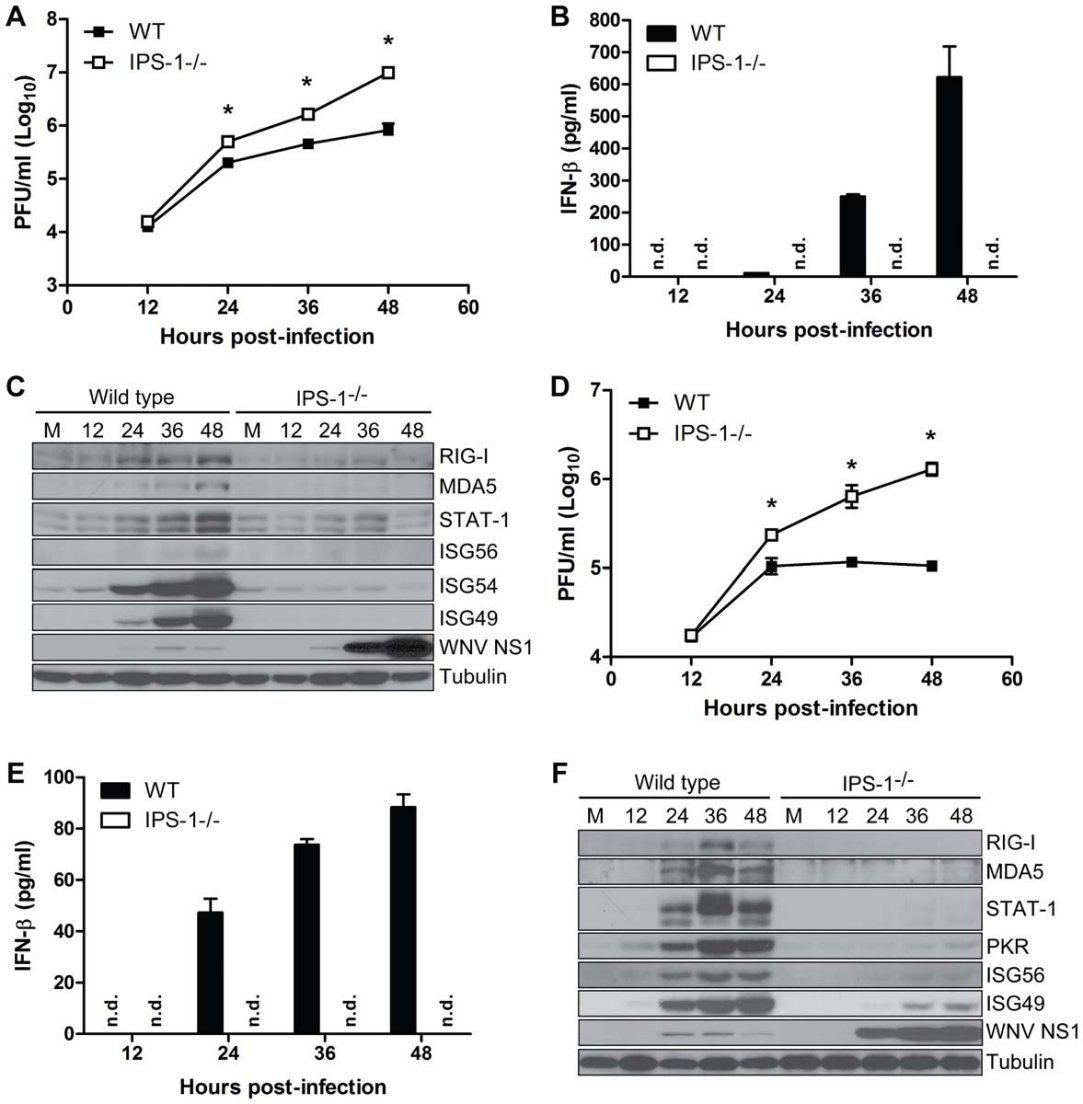
IPS-1 is essential for triggering the innate immune response to WNV infection and controlling virus replication in myeloid cells. Primary bone-marrow derived dendritic cells (A–C) and macrophages (D–F) recovered from wild type mice and IPS-1^−/−^ mice were mock-infected (M) or infected with WN-TX at an MOI of 1.0. Cells and culture media were harvested at the times indicated pi for determination of virus load (A, D) and IFN-β production (B, E), respectively. n.d. = not detected. Graphs show the mean +/− standard deviation from three independent experiments. Asterisk denotes p<0.05. (C,F) Immunoblot analysis of protein abundance in lysates from mock-infected (M) and WN-TX infected cells. For panel C, STAT-1 expression in dendritic cells was normalized at each timepoint to loading control and compared to mock (relative fold induction WT, KO at 12 hours 1.72, 0.7; 24 hours 2.6, 0.8; 36 hours 4.6, 1.1; 48 hours 9.9, 0.6).

Myeloid cells, including tissue and lymphoid DC and macrophages (Mφ), are among the first cells to encounter WNV during infection and thus function to restrict the spread of virus to distant tissues and the CNS [2]. To define the role of IPS-1 in controlling virus replication and innate immunity in myeloid cells, we analyzed WNV infection and host responses in primary bone marrow-derived DC and Mφ recovered from wild type and IPS-1^−/−^ mice. DC and Mφ were infected at an MOI of 1.0 (relative to viral plaque assay quantification of BHK-21 cells; see Methods) and evaluated for virus replication, IFN induction, and innate immune triggering of ISG expression (**Fig 2**). IPS-1^−/−^ DCs sustained significantly higher WNV replication at 36 and 48 hours pi compared to wild type infected cells (**Fig 2A**). WNV infection of wild type DCs induced IFN-β secretion but this response was completely abolished in IPS-1^−/−^ DCs (**Fig 2B**). The lack of IFN-β induction in IPS-1^−/−^ DCs correlated with a lack of ISG expression including RIG-I, MDA5, and STAT-1 (**Fig 2C**). In addition, expression of ISG54 and ISG49, which are direct IRF-3 target genes [30],[31], were not induced during WNV infection of IPS-1^−/−^ DCs (**Fig 2C**). Moreover, ISG56, another IRF-3 target gene [31], was induced late during infection and to lower levels as compared to ISG54 and ISG49 in wild type, infected DCs. WNV infection of IPS-1^−/−^ Mφ resulted in significantly higher virus replication between 24 and 48 hours pi as compared to infected wild type cells (**Fig 2D**). Whereas wild type infected Mφ expressed IFN-β, this response was completely abolished in IPS-1^−/−^ Mφ (**Fig 2F**). We also observed a differential expression of ISGs and IRF-3-target genes within WNV-infected Mφ. RIG-I, MDA5, and STAT-1 were not induced in IPS-1^−/−^ Mφ, whereas, ISG56, ISG49, and PKR were expressed at reduced levels and with delayed kinetics. These data establish that IPS-1-dependent RLR signaling is the major innate immune signaling pathway that controls virus replication in conventional DCs and Mφ.

### The RLR signaling pathway triggers the innate immune response to WNV infection in primary cortical neurons

**Figure 3:**
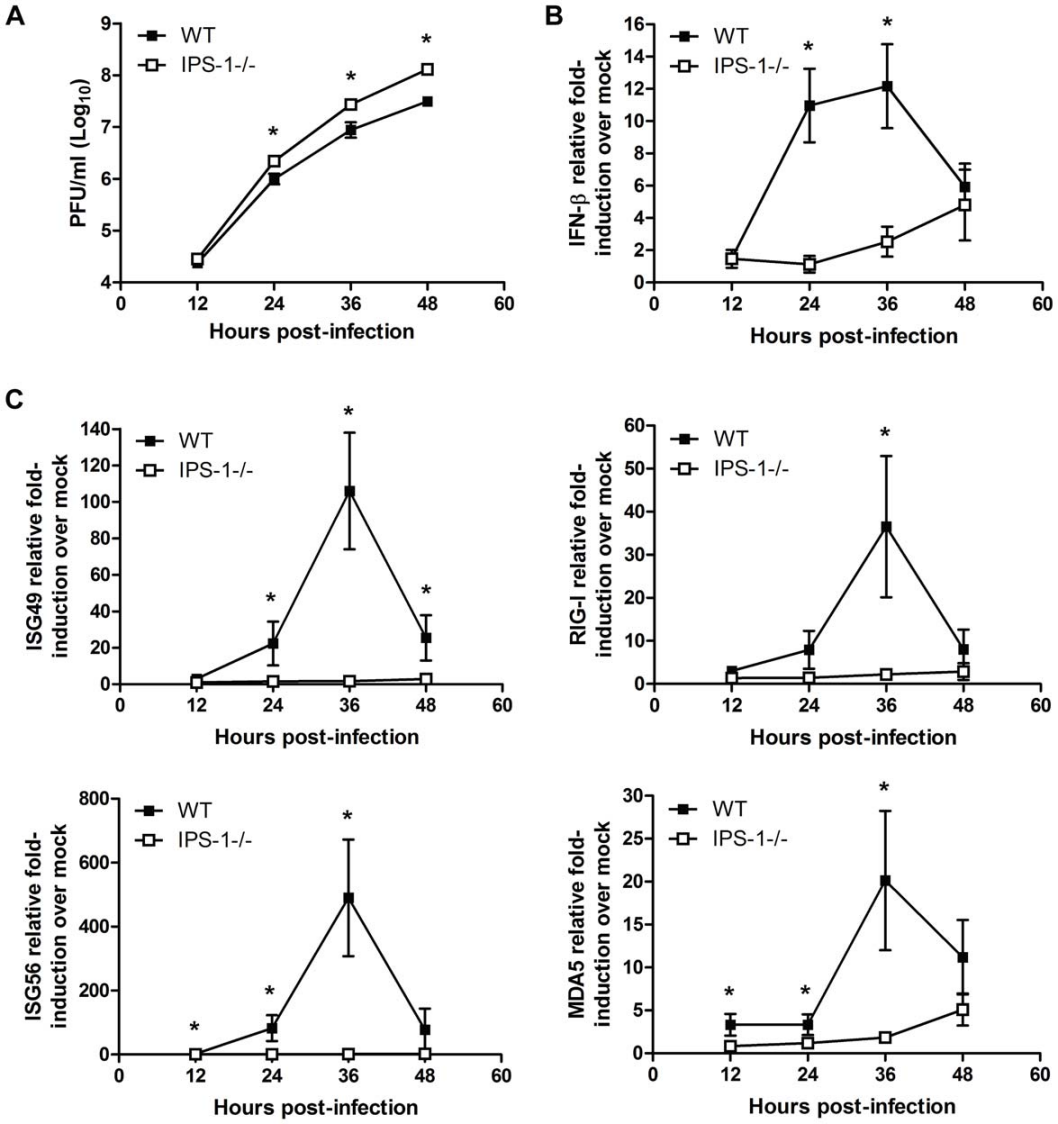
IPS-1 is essential for triggering innate immune defenses and controlling virus replication during WNV infection of primary cortical neurons. Primary cortical neurons (CN) from WT and IPS-1^−/−^ mice were infected at an MOI of 1.0. (A) Viral titers in the culture supernatants were determined by plaque assay. (B, C) mRNA expression was determined by RT-qPCR assay using primers-specific for (B) IFN-β or (C) ISG56, ISG49, RIG-I, and MDA5. Graphs show the mean +/− standard deviation from triplicate independent analyses. Asterisks denote p<0.05.

Neurons represent the target cell of WNV infection in the CNS and their death after infection is a key factor in pathogenesis and neurological sequelae [32],[33]. To define the role of RLR signaling in restricting virus replication in neurons, primary cortical neurons were generated from wild type and IPS-1^−/−^ mice. Cells were infected at an MOI of 1.0 with WN-TX and virus yield, IFN-β induction, and ISG expression were evaluated. In the absence of IPS-1, WNV replicated faster and to higher levels resulting in a 2.2 and 4.2-fold (p<0.05) increase in viral production at 24 hrs and 48 pi, respectively as compared to infected wild type neuronal cells (**Fig 3A**). This relatively modest virologic effect in neurons compared to that observed in IPS-1^−/−^ DC and Mφ was expected, as IFN-α or -β pre-treatment only inhibits WNV infection in cortical neurons to a maximum of 5 to 8-fold [12], suggesting that the IFN response is comparably less potent in neurons. IFN-β expression was induced to lower levels in IPS-1^−/−^ neurons compared to wild type infected neurons at 24 (10-fold, p<0.05) and 36 hours pi (5-fold, p<0.05) despite the higher levels of virus replication (**Fig 3A and 3B**). Expression of ISGs, (including RIG-I and MDA5) and IRF-3 target genes (including ISG56 and ISG49) followed this pattern and were dependent on IPS-1 for rapid and high level expression (**Fig 3C**). The presence of IFN-β and ISG transcripts in IPS-1^−/−^ cells at 48 hrs pi is consistent with the finding that TLR3 has an independent and subordinate role in triggering innate immune responses in cortical neurons at later time points after WNV infection [**23**]. These results demonstrate that the RLR signaling pathway controls virus replication and induces innate immune responses against WNV infection in cortical neurons.

### Neuronal destruction and CNS inflammation are enhanced in WNV infected IPS-1^−/−^ mice

**Figure 4:**
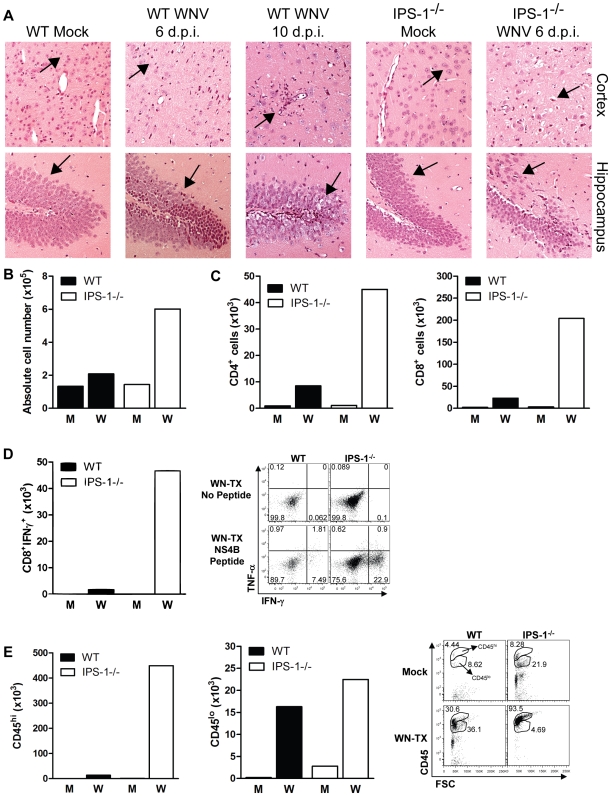
Increased CNS inflammation in WNV-infected IPS-1^−/−^ mice. (A) H&E stained saggital brain tissue sections. The arrows denote areas of interest. (B–E) Brain leukocytes were recovered from wild type and IPS-1^−/−^ mice six days pi. (B) The total number of brain lymphocytes was determined by cell counting. (C) Total CD4+ (left) and CD8+ T cells (right), (D) Total WNV-specific CD8+ T cells (left) with a representative analysis of the frequency of TNF-α and IFN-γ expression within brain CD8+ cells (right left), and (E) Total number of microglia/infiltrating macrophages (left) were determined by flow cytometry (right; representative analysis). M = Mock; W = WN-TX. Data are representative of two or more independent experiments, and each analysis represents a pool of 5 mouse brains.

To determine the role of the RLR pathway in protection of neurons against WNV pathogenesis *in vivo*, we conducted histological analysis of brain tissue from wild type and IPS-1^−/−^ mice infected with WN-TX (**Fig 4A**). Analysis of brain sections from infected wild type mice revealed little or no inflammation or neuronal damage, with sparse and focal cell infiltrates restricted to the hippocampus and cerebral cortex on day 6 pi. By day 10 pi (a timepoint in wild type mice in which peak virus replication in the CNS occurs [34]) cellular infiltrates were present in the parenchyma and neuronal destruction was observed throughout the cortex and hippocampus. In contrast, brain sections from infected IPS-1^−/−^ mice recovered on day 6 pi displayed extensive injury to neurons in the cortex and granular neurons of the hippocampus. Damaged neurons appeared pyknotic with vacuolation, degeneration and cell dropout. Somewhat surprisingly, we observed extensive inflammation in the brains from infected IPS-1^−/−^ mice within the cortex, hippocampus, and cerebellum (data not shown) displaying prominent perivascular and parenchymal immune cell infiltrates.

To evaluate the composition and antigen-specificity of the inflammatory cells within the brains of WNV-infected mice, lymphocytes were isolated from infected brains on day 6 pi and were characterized from pools (n = 5) of wild type and IPS-1^−/−^ infected mice. Brains from IPS-1^−/−^ infected mice showed an 2.9-fold increase in the total number of immune cells as compared to wild type infected mice (**Fig 4B**), and this was associated with an increase in absolute numbers of infiltrating CD4+ and CD8+ T cells (**Fig 4C**). Among the brain CD8+ T cells isolated from IPS-1^−/−^ mice, there was a remarkable 27-fold increase in the number of antigen-specific cells that expressed IFN-γ after treatment with an immundominant NS4B peptide (**Fig 4D**) [35],[36]. Analysis of microglia/Mφ cells, based on relative surface expression of CD45 and CD11b [37], revealed increased numbers of microglial cells (CD45+^lo^/CD11b+) and infiltrating macrophages (CD45+^hi^/CD11b+) within the brains of infected IPS-1^−/−^ mice when compared to wild type mice (**Fig 4E**). Similar findings were observed in the spinal cords from infected IPS-1^−/−^ mice (data not shown). Combined with the histological analysis, these results demonstrate that in the absence of IPS-1, WNV infection induces a strong inflammatory response in the CNS. While this response is likely associated with increased viral loads, the failure of this increased inflammatory response to elicit protection or control CNS pathology, in the absence of IPS-1, suggests a role for the RLR signaling pathway as a regulatory program that controls the quality of the inflammatory response to WNV infection.

### Serum cytokine levels

**Figure 5:**
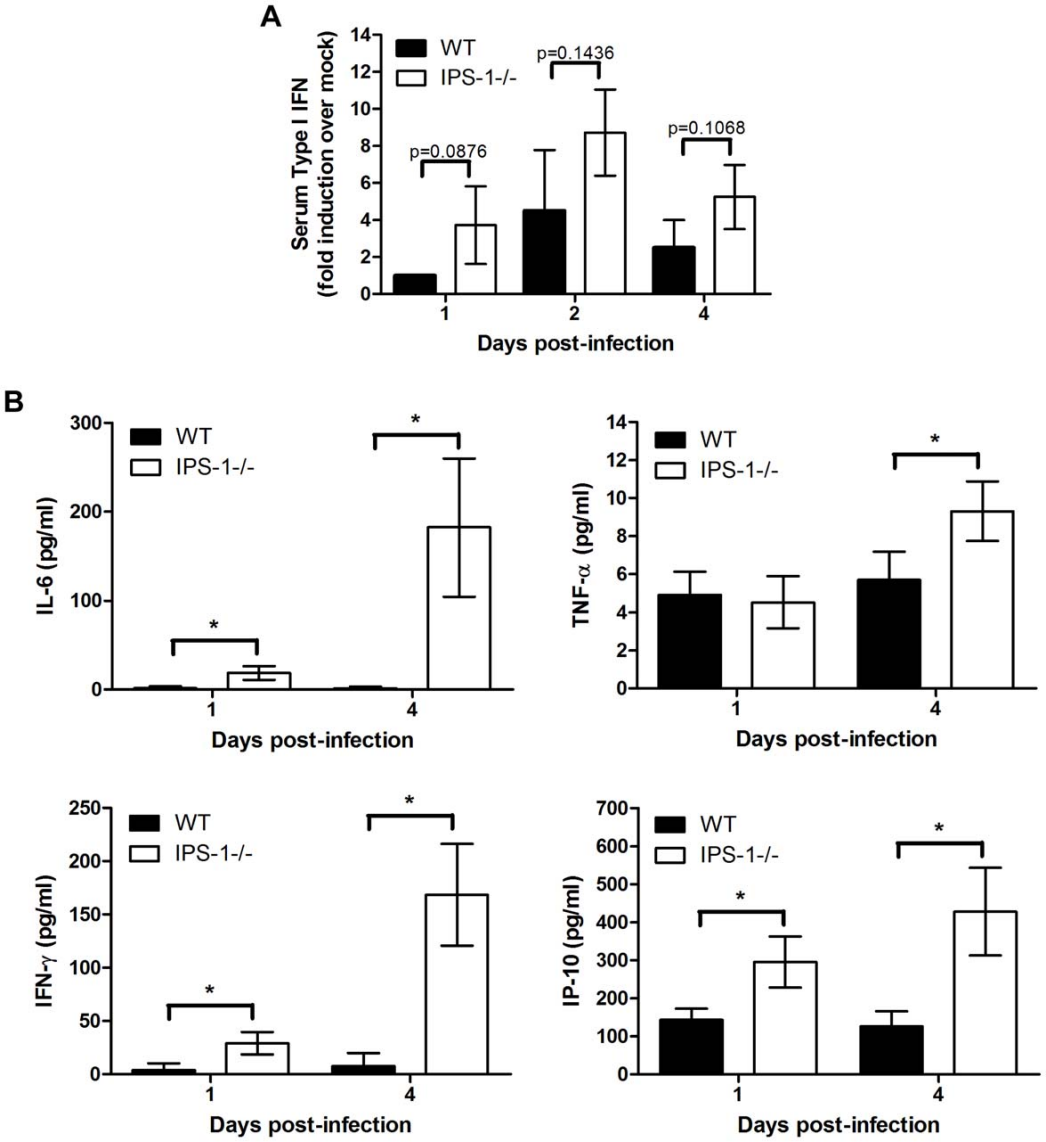
Enhanced levels of IFN, proinflammatory cytokines, and chemokines in serum from WNV-infected IPS-1^−/−^ mice. Serum was collected from wild type and IPS-1^−/−^ mice. (A) Type I IFN levels at 1, 2, and 4 days pi. (B) Proinflammatory cytokines and chemokines were measured on days 1 and 4 pi. Graphs show the mean +/− standard deviation from triplicate measurements of duplicate experiments. Asterisks denote p<0.001.

To further characterize how IPS-1 modulates the inflammatory response to WNV infection, we measured levels of systemic type I IFN, proinflammatory cytokines, and chemokines present in the serum of WNV-infected mice at 1 and 4 days pi. Paradoxically, a trend towards more rapid induction and increased levels of type I IFN were observed in the serum of IPS-1^−/−^ mice compared to wild type mice (**Fig 5A**). We note that in this case type I IFN was detected and quantified through a mouse-specific type I IFN bioassay, which does not differentiate between the IFN-α or -β species. This result is consistent with our recent studies showing that serum type I IFN levels accumulate during WNV infection in an IRF-7-dependent but IRF-3-independent manner [8],[9]. In this case IFN-α species are likely accumulating through a TLR7-dependent signaling pathway involving plasmacytoid DCs, which do not require the RLR pathway for IFN production [38]. More recently, Town et al. assessed the role of TLR7 and MyD88^−/−^ during WNV infection and found that mice lacking MyD88 produced elevated levels of systemic IFN during WNV infection [25]. Thus, during WNV infection systemic IFN is regulated through RLR-dependent and independent processes. Correspondingly, when compared to wild type mice, IPS-1^−/−^ infected animals, which show higher viremia (**Fig 1B**) produced increased serum levels of proinflammatory cytokines and chemokines in response to WNV infection. Elevated levels of systemic IL-6, TNF-α, CXCL10, and IFN-γ were observed at 1 and/or 4 days pi in IPS-1^−/−^ mice (**Fig 5B**). Serum cytokine levels were also compared between uninfected wild type and IPS-1^−/−^ mice and showed no differences in basal cytokine expression (data not shown). These results demonstrate that in the absence of IPS-1, greater proinflammatory cytokine and chemokine responses are induced during WNV infection.

### Altered WNV-specific antibody profiles in IPS-1^−/−^ mice

WNV-specific antibody responses are essential for suppressing viremia and virus dissemination and limiting lethal WNV infection [39],[40]. To determine if a deficiency in IPS-1 modulated the quality and quantity of the humoral immune response, we characterized the antibody profile in sera during WNV infection. In wild type mice, neutralizing virus-specific IgM antibodies are typically detectable by day 4 pi with WNV and production of neutralizing virus-specific IgG antibodies follow between days 6 and 8 pi [40]. A time course analysis in wild type and IPS-1^−/−^ infected mice showed that between 4 and 6 days pi, WNV-infected IPS-1^−/−^ mice exhibited significantly higher levels of virus-specific IgM, IgG, and IgG subclasses as compared to infected wild type mice (**Table 1**). WNV-specific IgG1 antibodies were detected at low levels on day 6 pi in sera from wild type and IPS-1^−/−^ mice. Additionally, we observed a ∼72.9-fold increase in WNV-specific IgG2a levels in infected IPS-1^−/−^ as compared to wild type mice on day 6 pi and ∼2.2-fold increase on day 8 pi. Assessment of the virus-specific antibody responses through a PRNT assay revealed that neutralization titers in sera from wild type mice increased dramatically between 6 and 8 days pi. Sera from IPS-1^−/−^ infected mice exhibited a modest increase in neutralization titer to 1∶1280, despite having much higher levels of virus-specific antibodies. This difference translated into a serum neutralization index that was ∼39-fold lower on day 6 pi in the infected IPS-1^−/−^ mice compared to wild type mice. These results demonstrate that the humoral responses in WNV-infected IPS-1^−/−^ mice are distinct from responses in wild type infected mice. Thus, RLR signaling and IPS-1 actions likely contribute to regulatory processes that govern the levels, IgG class switching, and neutralizing capacity of antibodies generated in response to WNV infection.

**Table 1:** WNV-specific antibody titers.

	Wild type			IPS-1^−/−^	
	Day 4	Day 6	Day 8	Day 4	Day 6
IgM^a^	<20	160±49	1620±0	47±20	1620±0
IgG^b^	60±0	520±575	12150±4860	40±22	102060±45174
IgG1^b^	<20	20±0	<20	<20	20±0
IgG2a^b^	<20	126±203	4050±1620	<20	9180±6033
PRNT_50_	<20	320±0	7040±3840	<20	1280±0
Neutralization Index^c^	<0.25	0.471	0.511	<0.23	.012

a IgM titers were determined by an arbitrary cutoff at an OD of 0.2 minus the value from mock infected mice.

b IgG, IgG1, and IgG2a titers determined by OD values that were greater than 3 standard deviations above background signal.

c Neutralization index was calculated by dividing PRNT_50_ titers by the total IgM^a^ plus IgG^b^ titer for each point.

### Enhanced inflammation in lymphoid organs associates with altered DC subsets and reduced numbers of regulatory T (T_reg_) cells in WNV infected IPS-1^−/−^ mice

**Figure 6:**
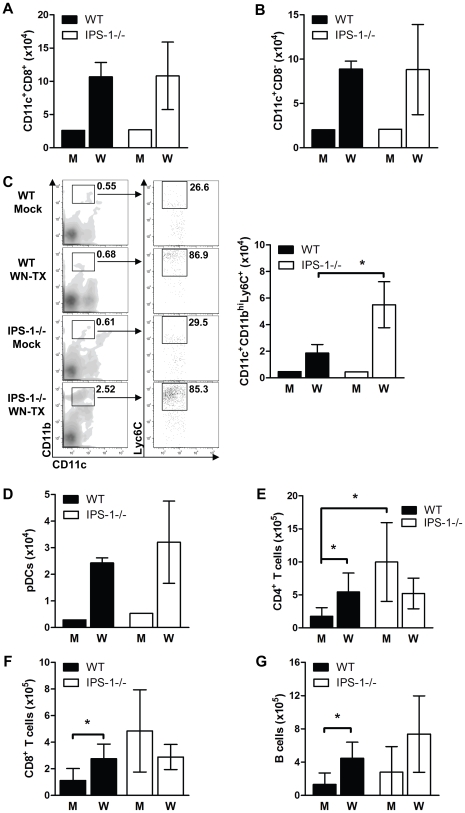
Immune cell subsets within the popliteal draining lymph node during acute WNV infection. Wild type and IPS-1^−/−^ mice were mock-infected (M) or infected with WN-TX (W), and popliteal draining lymph nodes were harvested 24 hr later. Lymphoid cells were isolated and cells were analyzed by flow cytometry. (A–C) total numbers of cells expressing various dendritic cell surface markers, (D) plasmacytoid cell surface markers (CD11c^int/lo^/B220+/siglec H+). (E and F) T cells (G) B cells. A representative flow cytometric analysis of the CD11c+/CD11b^hi^/Ly6C+ DC subset is shown in C (left panel set). Data show the mean +/− standard deviation from triplicate samples of duplicate experiments. Asterisks denote p<0.05.

To characterize the immune parameters associating with the dysregulated inflammatory and humoral responses in WNV infected IPS-1^−/−^ mice, we analyzed the immune cell composition in draining lymph node and spleen tissues. Wild type and IPS-1^−/−^ mice were challenged with diluent alone or with WN-TX, and draining popliteal lymph node (DLN) and the spleen were harvested at 1 and 6 days pi, respectively. Analysis of the popliteal DLN provides insight into how IPS-1 modulates the inflammatory response immediately after infection whereas assessment of the spleen elucidates characteristics of the adaptive immune response prior to the infection endpoint. Immune cells were isolated from the popliteal DLN and were characterized by flow cytometry (**Fig 6**). Analysis of CD8α DC subsets, which are phenotypically the major antigen presenting cells within lymphoid tissues and are implicated in eliciting virus-specific CD8 T cell in response to acute WNV infection [41], showed that infected wild type and IPS-1^−/−^ mice exhibited similar increases in the numbers of CD8α^+^ and CD8α^−^ DCs compared to mock-infected mice (**Fig 6A, B**). However, a significant increase (∼3-fold; p<0.05) of a proinflammatory DC subset, characterized as CD11c^+^CD11b^hi^Ly6C^+^, was observed within the popliteal DLNs of IPS-1^−/−^ infected mice (**Fig 6C**). This DC subset is monocyte-derived and typically recruited to sites of acute inflammation where they propagate the inflammatory response [42]. We found that these DC subsets were not significantly expanded and showed no differences in their recruitment to the DLN in either wild type or IPS-1^−/−^ infected mice at 12 hours pi (data now shown). Thus, as early as 24 hours pi, an elevated cellular inflammatory response is initiated in the IPS-1^−/−^ mice. In contrast, similar increases in plasmacytoid DCs were observed within infected wild type and IPS-1^−/−^ infected mice (**Fig 6D**), demonstrating that an absence of IPS-1 did not directly affect expansion and/or recruitment of this DC subset. Within the popliteal DLNs, mock-infected IPS-1^−/−^ mice compared to wild type mice generally showed elevated numbers of B cells, CD4+ T cells (p<0.05), and CD8+ T cells (**Fig 6E, F, and G**). These results suggest that IPS-1 contributes to the homeostasis of lymphocyte populations within LNs. WNV infection of wild type mice increased the number of B cells (3.4 fold), CD4^+^ T cells (3.1 fold), and CD8^+^ T cells (2.3 fold; p<0.05) in the DLN within 24 hours pi. Similar increases in B cells were observed upon infection of IPS-1^−/−^ mice. However, the number of CD4^+^ and CD8^+^ T cells was reduced in the DLN after WNV infection of IPS-1^−/−^ mice. Thus, in the absence of IPS-1, WNV infection specifically increases the number of inflammatory Ly6c^+^ DCs but suppresses the overall expansion and/or recruitment of T cells in the DLN.

**Figure 7:**
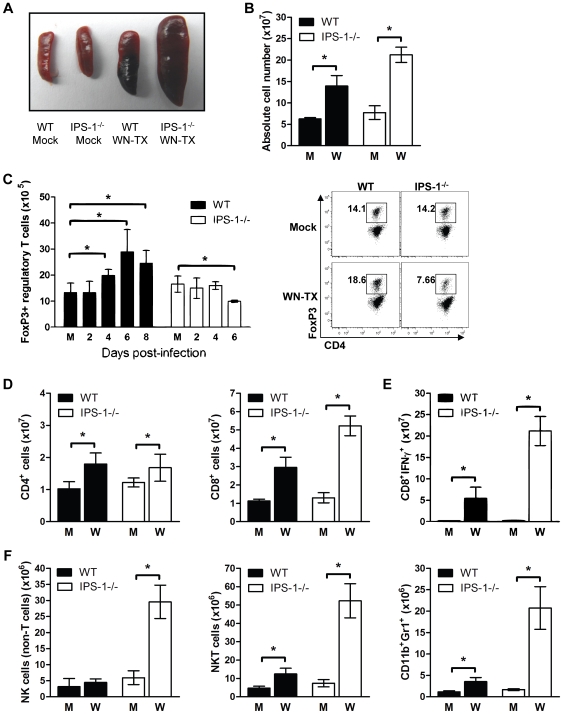
Enhanced inflammation in IPS-1^−/−^ infected mice associates with a lack of T_reg_ expansion during WNV infection. Wild type and IPS-1^−/−^ mice were mock-infected (M) or infected with WN-TX (W). Spleens were harvested and immune cells were isolated, counted, and characterized by flow cytometry. (A) Spleen morphology. (B) Absolute cell counts. (C) Total CD4+/FoxP3+ regulatory T cells (left; M = mock) and representative flow cytometric analysis of cell frequency from samples on day 6 pi (right). (D) CD4+ and CD8+ T cells. (E) NS4B antigen-specific CD8+ T cells. (F) CD4−/CD8−/NK1.1+ NK cells, CD4+/CD8+/NK1.1+ NKT cells, and CD11c+/Gr1+ neutrophils. Graphs show the mean +/− standard deviation from triplicate samples of duplicate experiments. Asterisks denote p<0.05.

We further analyzed the lymphocyte composition of the spleen on day 6 after WNV infection (**Fig 7**). Gross pathologic analysis revealed that WNV infection of IPS-1^−/−^ mice results in massive splenomegaly whereas infection of wild type mice induces only a slight increase in spleen size (**Fig 7A**). Cell analysis revealed increased numbers of total lymphocytes in the spleens of infected IPS-1^−/−^ mice as compared to wild type mice (**Fig 7B**).

Regulatory T (T_reg_) cells have recently been shown to contribute to the dampening of inflammation and adaptive immune responses during acute virus infections [26],[43],[44]. Moreover, a reduction in the number of circulating T_reg_ in mice leads to enhanced lethality after WNV infection [45]. A time course analysis of T_regs_ in wild type mice revealed that WNV infection results in a significant increase in the numbers of FoxP3+ T cells as compared to mock-infected mice beginning on day 4 and peaking by day 6 pi (**Fig 7C**), indicating the expansion of T_regs_ during acute WNV infection. Despite their marked increase in viral load, the infected IPS-1^−/−^ mice did not display an increase in the numbers of FoxP3+ T cells at any timepoint analyzed. Thus, IPS-1 signaling directly or indirectly impacts T_reg_ proliferation and does so independently of viral load.

We also observed that spleens from infected IPS-1^−/−^ mice exhibited significantly increased numbers of CD8+ T cells in comparison to those from infected wild type mice, whereas the expansion of splenic CD4+ T cells in wild type and IPS-1^−/−^ mice were not different (**Fig 7D**). The spleens from WNV-infected IPS-1^−/−^ mice showed significantly higher numbers (3.9-fold; p<0.05) of WNV-specific CD8+ T cells producing IFNγ.

To further define the phenotype associated with WNV-induced splenomegaly in IPS-1^−/−^ mice, we also assessed the numbers of NK cells and neutrophils. Spleens from infected IPS-1^−/−^ mice contained greater numbers of NK cells (CD4^−^CD8^−^NK1.1^+^cells), NKT cells (CD4+/CD8+/NK1.1+ cells) and neutrophils (CD11b^+^Gr1^+^ cells) (**Fig 7E**). Although WNV-infected wild type mice infected displayed slight increases in the absolute numbers of these specific cell types, a deficiency of IPS-1 resulted in a more marked enhancement of these immune cell populations.

## Discussion

In this study, we establish a major role for RLR signaling in protection from WNV pathogenesis, and demonstrate that IPS-1 is critical for the control of WNV infection *in vivo*. Our studies indicate that IPS-1-dependent RLR signaling functions to establish balanced, effective, and protective innate and adaptive immune responses, and that IPS-1 links adaptive immune regulation with the innate immunity triggered by RLR signaling during WNV infection. In the absence of IPS-1 *in vitro*, innate immune defense programs of myeloid DCs and macrophages were ablated or severely attenuated. Moreover, *in vivo* analysis of infected IPS-1^−/−^ mice showed altered IgG and IgM antibody responses with diminished virus neutralization activity. The inflammatory response to WNV infection is regulated by IPS-1-dependent processes such that a deficiency of IPS-1 resulted in elevated proinflammatory cytokine and chemokines and increased numbers of inflammatory DCs, antigen-specific T cells, natural killer cells, and neutrophils in lymphoid organs, and activated macrophage/microglial cells within the CNS. The dysregulated inflammatory response to WNV infection in IPS-1^−/−^ mice was associated with a reduction in the numbers of T_reg_ cells and their failure to expand during acute infection. These observations demonstrate the critical role of IPS-1 in mediating RLR signaling of innate antiviral immunity against WNV infection, and reveal novel features of IPS-1 function in regulating immune homeostasis, inflammation, and adaptive immunity to infection.

Although infection of primary DCs, macrophages, and neuronal cells failed to induce type I IFN upon WNV infection, WNV-infected IPS-1^−/−^ mice showed enhanced systemic type I IFN responses. This finding agrees with previous studies that indicate both IPS-1-dependent and -independent pathways contribute to the systemic type I IFN production *in vivo* [8],[9],[23],[25]. Most importantly, the enhanced tissue tropism and rapid viral entry into the CNS observed in the IPS-1^−/−^ mice is not affected by the elevated systemic IFN responses. This suggests that local tissue-specific and intracellular responses triggered by RLR-dependent signaling are more essential for reducing viral burden and dissemination. One possible explanation is that a distinct set of RLR-responsive genes function to control virus replication at the site of infection. This could explain, in part, the elevated levels of virus replication, enhanced tissue tropism and cell-to-cell spread in mice or cells deficient in IRF3 or IRF-7, each of which are downstream transcription factors of RLR signaling [8],[9],[10]. Additionally, it is likely that pDCs, which are specialized dendritic cells for producing systemic type I IFN during a viral infection [46], are likely contributing to the IFN responses observed during WNV infection. Studies by Silva et al. have shown that WNV triggers IFN induction in pDCs through a replication-independent manner [47]. Interestingly, within the DLN, we observed similar expansion of pDCs between wild type and IPS-1^−/−^ infected mice, yet at the same timepoint (24 hours pi), elevated systemic type I IFN responses were observed in IPS-1^−/−^ mice. This suggests two possibilities: 1) splenic pDCs or circulating pDCs are likely responding to the high levels virus in the serum from the IPS-1^−/−^ infected mice to produce IFN at 24 hours pi and/or 2) various other cell types that express TLR3 and/or TLR7 are responding to WNV infection and contributing to systemic IFN responses. Taken together, these studies indicate that RLR signaling and the actions of IRF-3/7 are important in triggering IFN production and ISG expression to limit WNV replication and spread, and that TLR signaling may impart additional, RLR-independent defenses that regulate immunity against WNV infection.

The production of and response to type-I IFN is a major linkage point between innate and adaptive immunity, as IFN-α and IFN-β sustain B cell activation and differentiation [48],[49],[50], expand antigen-specific CD8+ T cells [51],[52], CD4+ T cells [53], and activation of NK cells [54]. One of the most intriguing aspects of this study was the global alteration of the immune response elicited in the IPS-1^−/−^ mice, indicating that RLR signaling couples innate immunity with regulation of the adaptive immune response. Infection of IPS-1^−/−^ mice exhibited increased IgM and IgG WNV-specific antibodies, enhanced WNV-specific CD8+T cell response, and increased expansion of neutrophils, NK cells and NK-T cells. One trivial explanation for these differences is that there is an increased antigen load in the absence of IPS-1 and, as a result, enhanced virus-specific (e.g. CD8+ T cells, IgG and IgM antibodies) and nonspecific (e.g. Neutrophils, NK cells) responses. However, there are several key findings from this study that argue against these responses simply being attributed to higher antigen load: (1) In the absence of IPS-1, the CD4 and CD8 T cells, which are protective against WNV infection [34],[35],[36],[55],[56],[57],[58], were significantly enhanced in the peripheral and CNS compartments but failed protect against infection. One explanation for this observation is that the expansion and migration of CD4 and CD8 T cells to different tissues was itself uncontrolled, resulting in T cell-mediated pathology rather than T cell-mediated protection. (2) While the quantity of virus-specific IgM and IgG antibody responses were greatly enhanced in the absence of IPS-1, there was a marked reduction in antibody quality in terms of neutralization capacity. In contrast deficiencies in TLR3 or MyD88 (data not shown) did not alter virus-specific antibody responses and neutralization capacities. Collectively, these findings suggest that RLR-dependent signaling coordinates effective antibody responses during WNV infection through as yet undefined pathway. (3) While systemic IFN responses provide a link between innate and adaptive immune responses, our studies suggest that the PRR signaling pathways (RLR-dependent vs –independent) and levels of IFN production in combination with production other proinflammatory cytokines or chemokines regulate the quantity and quality of the immune response during virus infection. Thus, in the absence of IPS-1 signaling, infected conventional DCs or Mφ, two integral cell types in establishing adaptive immunity, likely do not produce IFN or the normal array and level of proinflammatory cytokines/ chemokines. Instead, IFN and other mediators may be strictly produced by infected or bystander cells during WNV infection, occurring with altered kinetics and magnitude, through TLR-dependent pathways, such as TLR3 and/or TLR7 [23],[25]. (4) In the absence of IPS-1, the enhanced expansion of Ly6C+ “inflammatory” DCs failed to limit early WNV replication and dissemination. This inflammatory DC subset migrates to sites of infection, secretes pro-inflammatory cytokines, and promotes CD8+ T cell expansion during a secondary virus infection, suggesting it sustains the effector T cell response [59]. Our data indicate that Ly6C+ DC recruitment and/or expansion is governed by IPS-1-dependent events of RLR signaling. Thus, the aberrant recruitment/expansion of these inflammatory DCs may contribute to immunopathogenesis and limit development of an effective immune response to control WNV virus infection. (5) The lack of T_reg_ expansion during WNV infection correlated with altered IFN levels, increased proinflammatory cytokines and chemokine levels, and an increased number and distribution of antigen-specific CD8+ T cells. These observations implicate an indirect or direct role for IPS-1 in regulating T_reg_ levels during WNV infection, and provide evidence that links a lack of T_reg_ expansion to immune dysregulation.

While their importance in autoimmunity is established [60], recent studies have implicated an integral role for T_regs_ in controlling inflammation and adaptive immune responses during acute virus infections [26],[43],[44]. During acute infection T_regs_ function to locally contain and control the immune response with the dual outcome of suppressing viral dissemination while decreasing the likelihood of immune-mediated pathology. In support of this model, infection studies with herpes simplex virus (HSV) and mouse hepatitis virus (MHV), two well established models of viral encephalitis, have demonstrated the importance of T_regs_ in limiting proinflammatory cytokine and chemokine responses to protect the CNS and enhance survival [26],[43]. Recent work also implicates T_regs_ in the control of WNV pathogenesis, wherein peripheral expansion of T_regs_ was associated with asymptomatic infection among WNV-infected blood donors but reduced T_reg_ levels associated with WNV disease [45]. Furthermore, these studies revealed that the conditional depletion of T_reg_ cells in mice results in enhancement of WNV virulence and expansion of antigen-specific CD8 T cells. Interestingly, from our studies, type I IFN does not appear to be the major contributor to T_reg_ expansion during WNV infection, as T_regs_ failed to expand in the IPS-1^−/−^ infected mice despite their elevated levels of systemic type IFN. These observations suggest that RLR signaling through IPS-1 provides essential signals that directly or indirectly impart the expansion of T_regs_ during WNV infection.

We propose that IPS-1 coordinates an innate/adaptive immune interface wherein IPS-1- signaling after RLR engagement regulates the quantity, quality, and balance of the subsequent immune response. The integrity of the innate/adaptive immune interface is central to the eliminating virus but also restricting immunopathogenesis and inflammation during infection. RLR signaling is essential for triggering the innate immune response to RNA viruses that cause human disease, including the influenza viruses, respiratory syncytial virus and other paramyxoviruses, picornaviruses, reoviruses, flaviviruses, and hepatitis C virus [14],[19],[22]. Thus, in addition to WNV, IPS-1-dependent RLR signaling will likely have a broad impact for the control of inflammation, immune response quality, and viral disease.

## Methods

### Cells and viruses

BHK21 and L929 cells were maintained in Dulbecco's modified Eagle medium (DMEM) supplemented with 10% fetal bovine serum (FBS), 2mM L-glutamine, 1 mM sodium pyruvate, antibiotic-antimycotic solution, and 1× nonessential amino acids (complete DMEM). WNV strain TX 2002-HC (WN-TX) was isolated by as previously described [11]. Working stocks of WN-TX were generated by a single round of amplification on Vero-E6 (ccl-81; ATCC) cells, and supernatants were collected, aliquoted, and stored at −80°C. Virus stocks were titered by a standard plaque assay on BHK21 cells as previously described [40].

### Mouse experiments

IPS-1^−/−^ (C57BL/6×129Sv/Ev) and their wild type littermate control mice have been published [38],[61] and were obtained as a generous gift from Dr. S. Akira (Osaka University, Osaka, Japan). Mice were genotyped and bred under pathogen-free conditions in the animal facility at the University of Washington. Experiments were performed with approval from the University of Washington Institutional Animal Care and Use Committee. The methods for mice use and care were performed in accordance with the University of Washington Institutional Animal Care and Use Committee guidelines. Age-matched six to twelve week old mice were inoculated subcutaneously (s.c.) in the left rear footpad with 100 PFU of WN-TX in a 10 µl inoculum diluted in Hanks balanced salt solution (HBSS) supplemented with 1% heat-inactivated FBS. Mice were monitored daily for morbidity and mortality.

### Viral tissue burden and quantification

For *in vivo* virus replication studies, infected mice were euthanized, bled, and perfused with 20 ml of phosphate-buffered saline (PBS). Whole brain, spinal cord, kidney, and spleen were removed, weighed, homogenized in 500ul of PBS, and titered by plaque assay.

### Primary cell isolation and infection

Bone-marrow derived DC and Mφ were generated as described previously [9]. Briefly, bone marrow cells from wild type and congenic deficient mice were isolated and cultured for 7 days in either RPMI-1640 supplemented with granulocyte-macrophage-colony stimulating factor, and interleukin-4 (Peprotech) to generate myeloid DC or in DMEM supplemented with macrophage colony stimulating factor (Peprotech) to generate Mφ. On day 7, DC or Mφ were infected with WN-TX at an MOI of 1.0 and at 12, 24, 36, and 48 hours post-infection (hpi), supernatants were collected for titration of viral burden by plaque assay on BHK21 cells and levels of IFN-β (described below). Cells were collected in parallel for western blot analysis. Cortical neurons were isolated from 15-day-old embryonic mice and cultured as described previously [62]. On day 6 of culture, neurons were infected with WN-TX at an MOI of 1.0 and at 12, 24, 36, and 48 hpi, supernatants were collected for virus titration by plaque assay on BHK21 cells and cells were collected for RNA analysis by RT-qPCR (described below).

### Western blot analysis

Cells were lysed in modified RIPA buffer (10mM Tris [pH 7.5], 150mM NaCl, 0.5% sodium deoxycholate, and 1% Triton X-100) supplemented with protease inhibitor cocktail (Sigma) and phosphatase inhibitor cocktail II (Calbiochem). Protein extracts (25 µg) were analyzed by immunoblotting as described previously [11]. The following primary antibodies were used to probe blots: mouse anti-WNV from the Center for Disease Control; rabbit anti-ISG56, rabbit anti-ISG54, rabbit anti-ISG49, kindly provided by Dr. G. Sen; mouse anti-PKR from Santa Cruz; rabbit anti-RIG-I and rabbit anti-MDA5 from IBL; mouse anti-tubulin from Sigma; and rabbit anti-STAT-1 from Cell signaling. Secondary antibodies included peroxidase-conjugated goat anti-rabbit, goat anti-mouse, donkey anti-rabbit, and donkey anti-mouse were from Jackson Immunoresearch.

### RNA extraction and analysis

For analysis of viremia, serum was separated (BD Microtainer tube SST) and RNA was extracted as previously described [8]. WNV RNA copy number was measured by RT-quantitative PCR (RT-qPCR) as previously described [63]. For cultured cells, total RNA was extracted using the RNeasy kit (Qiagen), DNase treated (Ambion) and evaluated for ISG49, ISG56, IFN-β, RIG-I, and MDA5 RNA expression by one-step SYBR Green RT-qPCR. Specific primer sets for ISG-49, ISG-56, RIG-I, and IFN-β have been described previously [30],[64]. Primer sets for MDA5 are: 5′-GTGGTCGAGCCAGAGCTGAT and 3′- TGTCTCATGTTCGATAACTCCTGAA.

### Interferon bioassay and ELISA

IFN-α and -β were measured in sera using a biological assay as previously described [65]. Briefly, L929 cells were seeded at 3×10^4^ cells/well in a 96 well plate one day prior to the addition of interferon standards or experimental samples. Mouse sera (diluted 1∶10 in L929 media) were treated with UV light for 20 minutes to eliminate residual virus. Duplicate sera samples were then added to the 96-well plates in two-fold dilutions along with a murine IFN-β standard. The following day, EMCV challenge virus was added to the cells in 50 µl/well at an MOI of 5.0. Twenty-four hours later, cytopathic effect was measured by a blinded scorer and IFN levels in the sera was calculated based on the IFN standard. IFN-β in cell culture supernatants was analyzed using mouse-specific ELISA kits from PBL Biomedical Laboratories according to the manufacturer's protocol.

### WNV-specific antibody analysis

WNV-specific IgM, total IgG, IgG1, and IgG2a levels were determined by an ELISA using purified recombinant E protein as previously described [55]. The neutralization titer of serum antibody was determined by using a previously described plaque reduction neutralization assay [40]. Briefly, sera samples from mock or WN-TX infected mice were diluted in DMEM followed by incubation at 56°C for 30 minutes to inactivate virus and complement factors. Sera were further diluted in two-fold increments and incubated with 100 PFU of WN-TX at 37°C for 1 hour. Standard plaque assays were performed on BHK21 cells and the dilution at which 50% of plaques were neutralized was determined by comparing the number of plaques formed from WNV-infected sera samples to mock infected sera samples.

### Cytokine/chemokine analysis

WNV infected sera were analyzed for the presence and levels of TNF-α, IFN-γ, CXCL10 (IP-10), and IL-6 by a mouse-specific cytokine/chemokine Milliplex ELISA (Millipore).

### Histological analysis

Mock-infected or WNV-infected mice were exsanguinated and perfused with PBS, 4% paraformaldehyde, pH 7.3. Brains were embedded in paraffin and 10-µm sections were prepared and stained with hematoxylin and eosin (H&E) by the UW histology pathology laboratory. Sections were analyzed using a Nikon Eclipse E600 microscope (UW Keck microscope facility).

### Flow cytometric analysis

Draining lymph nodes from mice were isolated and digested with collagenase (Roche) and type I DNase in serum-free RPMI media at 37°C for 40 minutes with mechanical disruption. Cells were then incubated with RPMI media containing 10% FBS with EDTA and HEPES for 10 minutes at room temperature, pelleted, and resuspended in PBS containing 2% FBS and 0.1% sodium azide (FACS Staining buffer). Splenocytes were isolated, washed, and re-suspended in RPMI 1640 containing 10% FBS before *in vitro* stimulation. Cells were washed twice before FACS staining. For isolation of CNS immune cells, mice were euthanized and perfused extensively with PBS to remove residual intravascular leukocytes. Brains and spinal cords from 5 mice per experimental group were isolated and pooled. Tissues were minced in RPMI media, triturated, and digested with Liberase (Roche) and type I DNase in serum-free RPMI media at 37°C for 45 min. Immune cells were isolated after gradient centrifugation from a 37/70% Percoll interface and washed twice with FACS staining buffer. Immune cells were stained with antibodies specific to CD11c, CD11b, B220, CD3, CD25, CD4, CD8, NK1.1, Gr-1, siglec H, and CD45 (all reagents from eBiosciences). Intracellular FoxP3 staining was performed as previously described [26]. Intracellular IFN-γ staining was performed on splenocytes and CNS immune cells as previous described [35],[36]. Briefly, lymphocytes were stimulated with 1 µg/ml of the WNV NS4B peptide (SSVWNATTAI) for 4 h at 37°C. Cells were washed and stained for cell surface markers followed by permeabilization-fixation using the Cytofix-Cytoperm Kit (BD-PharMingen) and stained with a Pacific-Blue conjugated IFN-γ antibody (eBiosciences) at 4°C for 30 min, washed and analyzed by flow cytometry. Flow cytometry was performed on a BD LSRII machine using BD FACSDiva software. Cell analysis was performed on FlowJo (v.8.7.2) software.

### Statistical analysis

For *in vitro* studies and immune cell analysis an unpaired student T-test was used to determine statistical differences. For *in vivo* viral burden analysis, Mann-Whitney analysis was used to determine statistical differences. Kaplan-Meier survival curves were analyzed by the log-rank test. A *p*-value≤0.05 was considered significant. All data were analyzed using Prism software (GraphPad Prism5).
